# The LARGE Principle of Cellular Reprogramming: Lost, Acquired and Retained Gene Expression in Foreskin and Amniotic Fluid-Derived Human iPS Cells

**DOI:** 10.1371/journal.pone.0013703

**Published:** 2010-10-29

**Authors:** Katharina Wolfrum, Ying Wang, Alessandro Prigione, Karl Sperling, Hans Lehrach, James Adjaye

**Affiliations:** 1 Molecular Embryology and Aging Group, Department of Vertebrate Genomics, Max Planck Institute for Molecular Genetics, Berlin, Germany; 2 Institute of Chemistry and Biochemistry, Department of Biology, Chemistry, and Pharmacy, Freie Universität Berlin, Berlin, Germany; 3 Institute of Human Genetics, Charité - Universitätsmedizin Berlin, Berlin, Germany; 4 Stem Cell Unit, Department of Anatomy, Medical College, King Saud University, Riyadh, Saudi Arabia; The University of Hong Kong, Hong Kong

## Abstract

Human amniotic fluid cells (AFCs) are routinely obtained for prenatal diagnostics procedures. Recently, it has been illustrated that these cells may also serve as a valuable model system to study developmental processes and for application in regenerative therapies. Cellular reprogramming is a means of assigning greater value to primary AFCs by inducing self-renewal and pluripotency and, thus, bypassing senescence. Here, we report the generation and characterization of human amniotic fluid-derived induced pluripotent stem cells (AFiPSCs) and demonstrate their ability to differentiate into the trophoblast lineage after stimulation with BMP2/BMP4. We further carried out comparative transcriptome analyses of primary human AFCs, AFiPSCs, fibroblast-derived iPSCs (FiPSCs) and embryonic stem cells (ESCs). This revealed that the expression of key senescence-associated genes are down-regulated upon the induction of pluripotency in primary AFCs (AFiPSCs). By defining distinct and overlapping gene expression patterns and deriving the LARGE (Lost, Acquired and Retained Gene Expression) Principle of Cellular Reprogramming, we could further highlight that AFiPSCs, FiPSCs and ESCs share a core self-renewal gene regulatory network driven by OCT4, SOX2 and NANOG. Nevertheless, these cell types are marked by distinct gene expression signatures. For example, expression of the transcription factors, *SIX6*, *EGR2*, *PKNOX2*, *HOXD4*, *HOXD10*, *DLX5* and *RAXL1*, known to regulate developmental processes, are retained in AFiPSCs and FiPSCs. Surprisingly, expression of the self-renewal-associated gene *PRDM14* or the developmental processes-regulating genes *WNT3A* and *GSC* are restricted to ESCs. Implications of this, with respect to the stability of the undifferentiated state and long-term differentiation potential of iPSCs, warrant further studies.

## Introduction

Human amniotic fluid cells (AFCs) represent a heterogeneous mixture of cells originating from different fetal tissues. They have been used for prenatal diagnosis of various congenital fetal abnormalities for more than fifty years [1]. Yet, especially within the last decade, molecular biology-based studies have revealed remarkable features of distinct subpopulations within bulk AFCs. For instance, in 1999, activity of the telomere-elongating enzyme telomerase was detected in young AFCs, with decreasing activity in aged AFCs [2]. Later on, the presence of cells exhibiting certain embryonic stem cell (ESC) features among bulk primary AFCs was reported [3], [4]. Other groups have demonstrated the existence of mesenchymal stem cells (MSCs) within the amniotic fluid [5]. Based on these observations, several strategies have been developed to sort stem cell-like populations out of bulk AFCs and different subpopulations have been characterized in more detail [6]–[10]. Multipotent properties [6], [7], [11], [12] and potential immune-privileged characteristics of particular AFCs [13], [14] support the idea of utilizing amniotic fluid as a source of fetal stem cells, with feasible application in regenerative medicine, especially in fetal tissue engineering approaches [13]. However, there are drawbacks associated with the use of AFCs for such purposes. For instance, primary cultures of bulk AFCs, like primary cell lines in general, senesce after prolonged culture periods *in vitro*. Besides, the fact that AFCs do not form teratomas *in vivo* [6], [15], [16] implies that not even the stem cell-like cells within the amniotic fluid are *bona fide* pluripotent cells. Hence, their ability to form complex, mature differentiated cell types may be restricted. Indeed, the capacity of AFCs to form specialized cell types and to contribute to the formation of certain tissues or organs *in vitro* and in xenotransplantation experiments *in vivo* is a subject of debate [6], [11], [17]–[23]. We believe that a means of assigning amniotic fluid cells greater value as an *in vitro* model system to investigate developmental processes, to conduct disease modeling, toxicological studies, drug research and exploitation in regenerative medicine could be achieved by cellular reprogramming of these cells to an undifferentiated ground state. As a result, AFCs acquire the ability to self-renew and become pluripotent.

The induction of pluripotency was first achieved in human somatic cells employing combinations of retroviral or lentiviral vectors encoding either *OCT4*, *SOX2*, *KLF4*, and *c-MYC* or *OCT4*, *SOX2*, *NANOG*, and *LIN28*, respectively [24], [25]. Since then, induced pluripotent stem cells (iPSCs) of different somatic origins, from healthy and diseased individuals have been generated using various techniques [26]–[28].

During the course of this study, the generation of iPSCs from human AFCs were described [15], [29], [30]. However, these studies failed to characterize amniotic fluid-derived iPSCs beyond the standard assays required to confirm induced pluripotency. Yet, for potential application of iPSCs in basic and applied research, various fundamental aspects of iPSCs, in general, and of this new AF-derived iPSC type, in particular, remain to be understood. Our study aimed at a more detailed molecular characterization of AFiPSCs. To this end, we generated AFiPSCs and demonstrated their ability to differentiate into the extraembryonic trophoblast lineage. This study also highlights the potential of cellular reprogramming to avert replicative senescence observed in bulk primary AFCs. Furthermore, we have analyzed similarities and differences between AFiPSCs, ESC lines H1 and H9 and fibroblast-derived iPSCs (FiPSCs) on the basis of global gene expression. We discuss a fundamental principle of cellular reprogramming, which we have coined LARGE, the Lost, Acquired and Retained Gene Expression principle. This refers to specific genes, which are either switched off, activated or which remain expressed upon induction of pluripotency. In this context, we demonstrate the activation of a common self-renewal and pluripotency-associated gene regulatory network upon cellular reprogramming. Furthermore, we highlight putative implications of the loss of distinct donor cell signature genes and the activation and/or retention of genes implicated in development processes upon cellular reprogramming.

## Materials and Methods

### Ethics Statement

Auxiliary samples of human AFCs obtained during routine amniocentesis were kindly donated by the clinical laboratory of Prof. Dr. Wegner/PD Dr. Stumm (Zentrum für Pränataldiagnostik, Kudamm-199, Berlin, Germany) after written informed consent. Utilization of these cells was approved by the ethics commission of the Charité Universitätsmedizin Berlin.

### Cell culture conditions

For the initial culture period (up to passage 5) AFCs were grown in AmnioMAX-C100 (Invitrogen, Carlsbad, CA, USA, www.invitrogen.com). Cells were subsequently cultured in alpha-MEM supplemented with 15% embryonic stem cell-qualified fetal bovine serum, 1% L-glutamine, 1% penicillin/streptomycin (all from Invitrogen), 18% Chang B and 2% Chang C (Trinova Biochem, Giessen, Germany, www.trinova.de) at 37°C in a humidified 5% CO_2_ atmosphere. AFCs were routinely passaged using 0.05% Trypsin/EDTA (Invitrogen) at varying ratios of 1∶3 or even 1∶6 when cells were sub-confluent.

Human ESC lines H1 and H9 (WiCell Research Institute, Madison, WI, USA, www.wicell.org), AFiPSCs (derived from human AFCs) and FiPSCs (derived from human neonatal foreskin fibroblasts, HFF1 (ATCC, #ATCC-SCRC-1041, Manassas, VA, USA, www.atcc.org)) were cultured under feeder-dependent and feeder-free conditions as described by Prigione et al. [31]. For comparative transcriptome analysis ESCs and AFiPSCs were adapted to feeder-free culture conditions in mTeSR (Stemcell Technologies, Vancouver, BC, Canada, www.stemcell.com).

### Retroviral production and iPSC generation

OCT4, SOX2, KLF4 and c-MYC retroviruses were generated using pMX vectors as described previously [24]. Briefly, 7.5*10(6) Phoenix Ampho Cells were seeded onto gelatin-coated T75 cell culture flasks and grown in DMEM (Invitrogen) supplemented with 10% FBS (Biochrome, Berlin, Germany, www.biochrom.de) for 16 h. The cells in one flask were then transfected with 12 µg of one of the retroviral DNA vectors encoding either *OCT4, SOX2, KLF4* or *c-MYC* using the FuGENE HD transfection reagent (Roche, Basel, Switzerland, www.roche.ch) according to the manufacturer's instructions. The retrovirus-containing medium was harvested 48 and 72 h post-transfection. For the generation of AFiPSCs, 180,000 AFCs were transduced with a cocktail of the respective retrovirus-containing media, supplemented with 4 µg/ml Polybrene (Sigma-Aldrich, Munich, Germany, www.sigmaaldrich.com) at a rate of 1.25 or 2.5 MOI on days 1 and 2 after plating. Each time, directly after the addition of retroviruses, the plates were centrifuged at 800×g, at 37°C for 99 min before replacement of the infectious medium by fresh medium (DMEM/10% FBS). The next day, the infected cells were plated onto irradiated MEFs on Matrigel-coated dishes in DMEM/10% FBS. Another 24 h later, the medium was switched to ESC medium [31] for a total period of 10 d, with replacement on alternate days. Afterwards, the infected cells were grown in mouse embryonic fibroblast-conditioned medium (MEF-CM) [32], which was changed at an interval of 2 d until reprogrammed AFiPSC colonies were manually picked 24 d post-transduction and expanded under ESC conditions. Currently, we have AF-derived iPSC lines 4, 5, 6, 10, and 41 in culture passaged more than 25 times (P25).

The generation of FiPSCs used for the comparative transcriptome analysis has been described [31].

### *In vitro* and *in vivo* differentiation of AFiPSCs

For *in vitro* differentiation, embryoid body (EB) formation of AFiPSC lines 4, 5 and 41 was induced in ESC medium without bFGF supplementation using the hanging-drop method [33]. After 2 to 3 d, EBs were placed onto low-attachment dishes. A week later, EBs were plated onto gelatin-coated dishes, allowed to differentiate for an additional 10 to 14 d and then stained. *In vivo* differentiation experiments were performed by EPO-Berlin GmbH (Germany, www.epo-berlin.de). Basically, approximately 2*10(6) cells of the AFiPSC lines 4 and 41 were collected by type IV collagenase-treatment or 0.05% Trypsin/EDTA-treatment, washed, pooled and injected s.c. into NOD.Cg-Prkdcscid Il2rgtm1Wjl/SzJ mice, commonly known as NOD scid gamma (NSG). Teratomas were collected approximately 63 d after injection and processed according to standard procedures for paraffin embedding and hematoxylin and eosin staining. Histological analysis was performed by a pathologist.

### Trophoblast differentiation of AFiPSCs

To induce differentiation into the trophoblast lineage, AFiPSC lines 5 and 41 were transferred onto Matrigel-coated cell culture dishes and grown in MEF-CM including 8 ng/ml bFGF (PeproTech, Rocky Hill, NJ, USA, www.peprotech.com) until they attained about 30 to 50% confluency. At this point, medium was changed to defined N2B27 medium (Invitrogen) lacking bFGF but including either 100 ng/ml BMP2 (PeproTech) or BMP4 (R&D Systems, Minneapolis, MN, USA, www.rndsystems.com) for a period of five days or a combination of 10 ng/ml BMP4 and 10 µM SB431542 (a TGFβRI inhibitor, Sigma-Aldrich) for 7 days. Undifferentiated controls were grown in N2B27 including 20 ng/ml bFGF only. After a period of 5 d or 7 d, including daily replacement of media, the cells were harvested for RNA isolation for qRT-PCR and global gene expression profiling analyses or fixed for immunofluorescence microscopy analysis.

### DNA fingerprinting and karyotyping

The origin of AFiPSC cell lines 4, 5, 6, 10, and 41 was confirmed by fingerprinting analysis, as previously described [34]. The primer pairs D17S1290 and D21S2055 were used; sequences are provided in Table S1. For the detection of probable karyotypic abnormalities in AFiPSC lines 4, 5, 6, and 41, chromosomal analysis was performed after GTG-banding at the Human Genetic Center of Berlin. For each line, 25 metaphases were counted and 10 karyograms analyzed.

### Illumina bead chip hybridization and data analyses

Total RNA was isolated using the RNeasy Mini Kit (Qiagen, Germantown, MD, USA, www.qiagen.com). In each case, 500 ng RNA were used as input for the bead chip hybridization (Illumina, San Diego, CA, USA, www.illumina.com). Processing of samples and the conversion of raw data was previously described [31]. For correlation coefficient analysis and the generation of Venn diagrams, detected gene expression was defined by a Detection P-Value <0.01. To be considered as differentially expressed, genes had to be at least 1.5 fold up- or down-regulated in a group-wise comparison of all AFiPSC or FiPSC lines with either AFCs at passage 6 or 17 or fibroblasts (Fibs), respectively. Accordingly, the FDR-adjusted P-Value for differential gene expression had to be <0.05. Human senescence-associated genes were derived using the AmiGO Browser version 1.7 of the Gene Ontology database (http://www.geneontology.org, 28^th^ of March 2010) [35]. Functional annotation and enrichment analyses were done using the DAVID platform version 6.7 (http://david.abcc.ncifcrf.gov/home.jsp) [36], [37]. Illumina ProbeIDs were used as input against the background of the Homo Sapiens species; analyses were executed based on DAVID default parameter settings (19^th^ of April 2010).

### Quantitative real-time polymerase chain reaction and data analyses

Quantitative real-time PCR (qRT-PCR) was performed and analyzed using ABI PRISM SDS 2.1 software (Applied Biosystems, Foster City, CA, USA, www.applied biosystems.com) and Microsoft Excel as described elsewhere [31]. All primer sequences are provided in Table S1. For detection of pluripotency-associated genes in AFiPSCs, the undifferentiated ESC line H1 was used as a positive control. For the analysis of BMP2- or BMP4-induced trophoblast differentiation of AFiPSCs, placental RNA (Clontech, Mountain View, CA, USA, www.clontech.com) was used as a positive control. Data are presented as mean LOG2 ratios with respect to biological controls and standard deviation.

### Immunofluorescence, alkaline phosphatase and cellular senescence staining

For the identification of ESC markers in undifferentiated AFiPSCs (lines 4, 5, 6, 10 and 41) and detection of lineage markers in AFiPSCs differentiated *in vitro*, cells were fixed, permeabilized and stained for immunofluorescent imaging as described by Prigione et al. [31]. The list of primary and secondary antibodies used is provided in Table S2. Nuclei were counter-stained with DAPI (100 ng/ml, Vector Laboratories, Burlingame, CA, USA, www.vectorlabs.com).

Alkaline phosphatase (AP) staining of all manually picked AFiPSC lines was performed following the manufacturer's instructions (Millipore, Billerica, MA, USA, www.millipore.com).

For staining of senescent cells, the Senescence beta-Galactosidase Staining Kit (Cell Signaling, Danvers, MA, USA, www.cellsignal.com) was used according to the manufacturer's protocol. All stainings were visualized and images were acquired using the confocal microscope LSM 510 Meta (Carl Zeiss, Oberkochen, Germany, www.zeiss.de). Processing of images was done with the help of AxioVision V4.6.3.0 (Zeiss) and Adobe Photoshop CS version 8.0 (Adobe, Munich, Germany, www.adobe.com) software.

## Results

### Senescence is bypassed by the derivation of AFiPSCs from human AFCs

**Figure 1:**
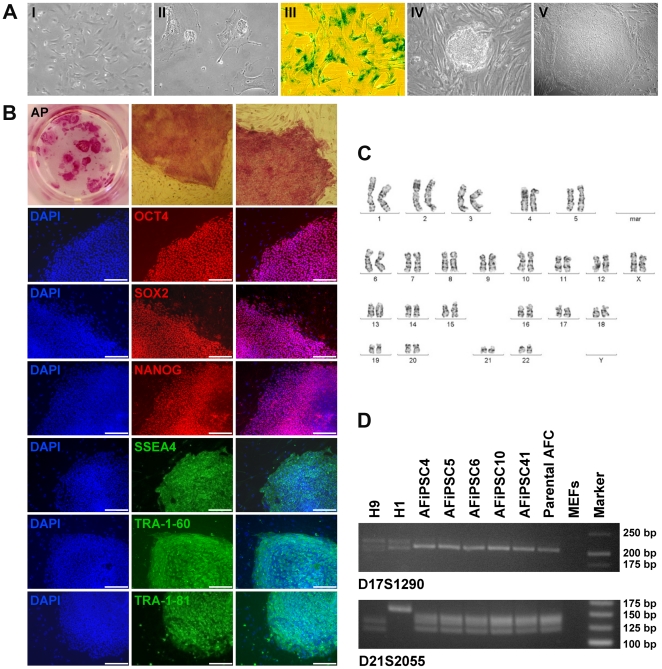
Senescence is bypassed by the derivation of human AFiPSCs from AFCs. (A-I) Primary human AFCs at passage 5, used for reprogramming. (A-II,-III) The same line at passage 17: Senescence is indicative by a striking morphological change (A-II) and beta-galactosidase staining (A-III). (A-IV) Colonies of AFiPSCs ten days post-transduction. (A-V) The morphology of a typical AFiPSC colony is indistinguishable from ESC colonies (scale bar  = 200 µm). (B) Top panel: AFiPSC colonies stained for alkaline phosphatase (AP) at passage 1. 2^nd^ to 7^th^ panel: Immunocytochemistry for the human nuclear ESC pluripotency markers OCT4, SOX2 and NANOG and for ESC surface antigens SSEA4, TRA-1-60 and TRA-1-81 (scale bars  = 200 µm). (C) AFiPSCs retain a normal karyotype after cellular reprogramming (mar  =  minimal altered region). (D) DNA fingerprinting verified the AFC origin of AFiPSC lines, excluding cross-contamination with ESC lines H1 and H9.

Under routine cell culture conditions, bulk primary human AFCs (Figure 1A-I) senesce at approximately passage 17 as determined by decelerated proliferation. These cells also have an enlarged and flattened morphology and stain positive for the senescence-associated beta-galactosidase (Figure 1A-II, -III). To bypass senescence and to enhance proliferation capacities of AFCs, we derived iPSCs from primary bulk AFCs by transduction with a retroviral cocktail consisting of OCT4, SOX2, KLF4 and c-MYC (OSKM) [24]. The resulting AFiPSC colonies appeared about seven days post-transduction (Figure 1A-IV), which is approximately two weeks earlier than what we and others have observed for fibroblast-derived iPSCs [24], [31]. Five clonal AFiPSC lines were expanded under ESC conditions and partly characterized. Of these, two lines underwent complete characterization. AFiPSCs were indistinguishable from ESCs (e.g. ESC line H1) in terms of morphology (Figure 1A-V) and proliferation. These AFiPSCs also resembled ESCs with respect to alkaline phosphatase (AP) activity and expression of several markers of the undifferentiated state, including NANOG, OCT4, SOX2, SSEA4, TRA-1-60, TRA-1-81 as determined by immunocytochemistry (Figure 1B). The AFiPSCs exhibited a normal karyotype several passages after their generation (Figure 1C) and their genetic relatedness to primary AFCs cells was confirmed by DNA fingerprinting analysis (Figure 1D).

### Pluripotency and *in vitro* and *in vivo* differentiation of AFiPSCs

**Figure 2:**
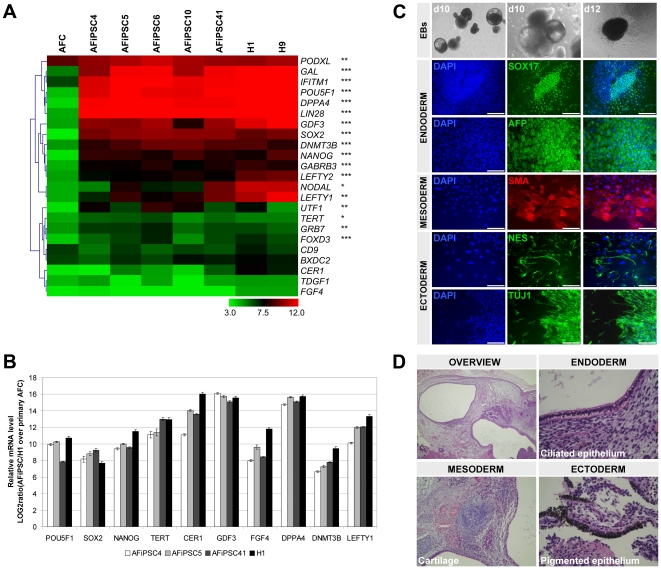
Pluripotency and *in vitro* and *in vivo* differentiation of AFiPSCs. (A) Microarray-derived gene expression levels of pluripotency-associated genes. Significantly up-regulated gene expression in AFiPSCs compared to AFCs is indicated by asterisks (*) FDR-adjusted P-Value <0.05, **) FDR-adjusted P-Value <0.01, ***) FDR-adjusted P-Value <0.001). The heatmap is colored by LOG2 average expression signals according to the color key on the bottom. (B) qRT-PCR for most commonly used pluripotency genes in AFiPSCs and ESC line H1. Bars and error bars represent LOG2 ratios (AFiPSCs or H1 relative to AFCs, respectively) and standard deviation. (C, D) AFiPSCs have the capacity to differentiate into derivatives representative of the three embryonic germ layers. (C) Embryoid body (EB)-based differentiation of AFiPSCs into various lineages *in vitro* as confirmed by immunofluorescent stainings of distinct germ layer marker proteins (lower panels); SOX17, alpha-fetoprotein (AFP); smooth muscle actin (SMA); nestin (NES) and class III beta-tubulin (TUJ1) (scale bars  = 200 µm). (D) Histological structures of ectodermal, mesodermal and endodermal lineages observed in teratomas.

Microarray-based transcriptional analysis revealed up-regulation of self-renewal and pluripotency-associated genes [38]–[40] in AFiPSCs in contrast to primary AFCs (Figure 2A). qRT-PCR validations, performed for a selection of these pluripotency-associated genes, confirmed the array-derived data (Figure 2B). The ability of AFiPSCs to differentiate into derivatives representative of all three embryonic germ layers was assessed by embryoid body differentiation *in vitro* (Figure 2C) and teratoma formation *in vivo* (Figure 2D). Markers or histological structures representing endoderm-, mesoderm- and ectoderm-derived lineages were detected in both assays (Figure 2C, D).

### Trophoblast differentiation of AFiPSCs

**Figure 3:**
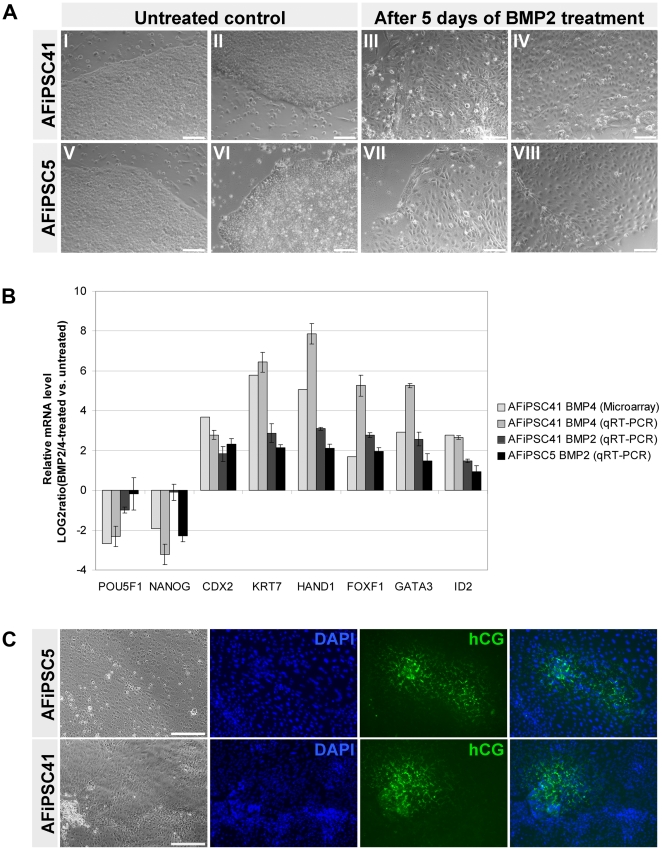
Trophoblast differentiation of AFiPSCs. Exogenous activation of BMP signaling cascades and blocking of TGFbeta/Activin/Nodal signaling results in differentiation of AFiPSCs into the trophoblast lineage. (A) When AFiPSCs were treated with 100 ng/ml BMP2 or BMP4 for five days, a morphological change from densely packed colonies (A-I, -II, -V, -VI) towards cobblestone-like cell clusters (A-III, -IV, -VII, -VIII) was observed when compared to the undifferentiated cells (scale bars  = 20 µm). (B) qRT-PCR and gene expression profiling (microarray) revealed down-regulation of pluripotency markers *POU5F1* and *NANOG*, but up-regulation of trophoblast markers *CDX2*, *KRT7*, *HAND1*, *FOXF1*, *GATA3* and *ID2* upon BMP2- or BMP4-treatment of AFiPSCs. Data are presented as LOG2 ratios (BMP-treated versus untreated AFiPSCs) and standard deviation. (C) Immunofluorescence-based detection of the placental hormone human chorionic gonadotropin (hCG) in AFiPSCs after treatment with 10 ng/ml BMP4 and 10 µM SB431542 over a period of seven days (scale bars  = 200 µm).

To test if AFiPSCs, like ESCs, can undergo trophoblast differentiation [41]–[44], we stimulated two AFiPSC lines with 100 ng/ml BMP2 or BMP4 over a period of five days. As a result, a morphological change from densely packed ESC-like colonies (Figure 3A-I, -II, -V, -VI) towards more loosely packed clusters of enlarged cells with typical cobblestone-like appearance (Figure 3A-III,-IV,-VII,-VIII) was observed. This is a characteristic feature of trophoblast differentiation of ESCs [41], [42]. Gene expression profiling and qRT-PCR analyses revealed down-regulation of the key pluripotency markers *POU5F1* and *NANOG* and up-regulation of the trophoblast markers *CDX2*, *KRT7*, *HAND1*, *FOXF1*, *GATA3*, and *ID2* (Figure 3B). Both, BMP2 and BMP4, induced similar effects, however, BMP4 was more efficient. When we treated the AFiPSCs with a combination of 10 ng/ml BMP4 and 10 µM SB431542 over a period of seven days, the same morphological changes could be observed and human chorionic gonadotropin (hCG), a hormone secreted by trophoblastic cells of the placenta, was detected by immunofluorescence microscopy analysis (Figure 3C).

### Global gene expression analyses of AFCs, AFiPSCs and ESCs

**Figure 4:**
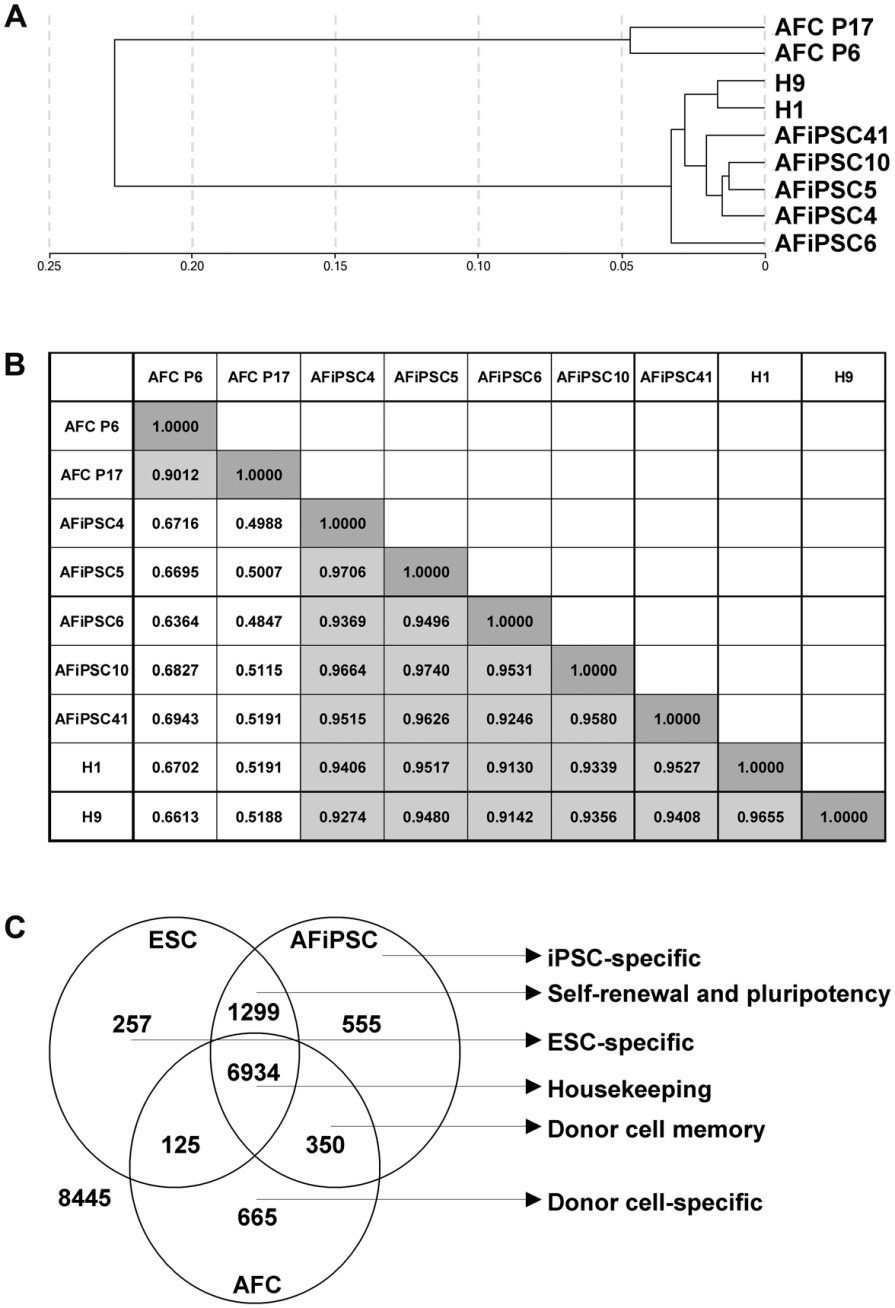
Global gene expression analyses of AFCs, AFiPSCs and ESCs. Transcriptome analyses of AFCs, AFiPSCs, ESCs (H1 and H9) using Illumina bead chips. (**A**) Hierarchical clustering based on detected gene expression using Pearson's correlation. (B) Table showing linear correlation coefficients (R^2^) between samples. Low R^2^ values (around 0.67) were detected between AFCs and AFiPSCs. ESCs and AFiPSCs exhibited high R^2^ values (around 0.94). (C) Venn diagram based on detected genes in AFCs, AFiPSCs and ESCs portraying distinct and overlapping transcriptional signatures between the different cell types.

Transcriptomes were profiled employing the Illumina BeadStudio platform to investigate the relatedness between primary AFCs, AFiPSCs and the ESC lines H1 and H9. Hierarchical clustering (Pearson's correlation) as well as linear correlation coefficient analysis based on expression signals of detected genes revealed similar, though not identical, transcriptomes of AFiPSCs and ESCs (average linear R^2^≈0.94). In contrast, the AFiPSC transcriptomes are distinct from those of primary AFCs at passage 6 (average linear R^2^≈0.67) and passage 17 (average linear R^2^≈0.50) (Figure 4A, B). The same analysis was repeated for separate replicate samples of HFF1-derived FiPSCs generated in our laboratory and ESC lines H1 and H9 [31]. This resulted in an average linear correlation coefficient of R^2^≈0.87, reflecting heterogeneity of iPSCs of different somatic origins. A Venn diagram was generated to highlight overlapping and distinct gene expression patterns in AFCs versus AFiPSCs and ESCs. We identified gene signatures representative of cellular housekeeping functions (6934 genes, e.g. *GAPDH*, *ACTB*, *PGK1*, *LDHA*), self-renewal and pluripotency (1299 genes, e.g. *POU5F1*, *SOX2*, *NANOG*, *LIN28*), a donor cell memory (350 genes, e.g. *KRT7*, *RGS7*), ESC-specificity (257 genes, *e.g. PRDM14*, *GSC*, *WNT3A*), donor cell (AFCs)-specificity (665 genes, e.g. *OXTR*, *HHAT*, *RGS5*, *NF2*, *CD59*, *TNFSF10*, *NT5E*) and an iPSC (AFiPSC)-specific gene expression signature (555 genes, e.g. *CNTFR*, *SIX6*) (Figure 4C, the entire gene lists are presented in Table S3).

### Expression of senescence-associated genes in primary AFCs and AFiPSCs

**Figure 5:**
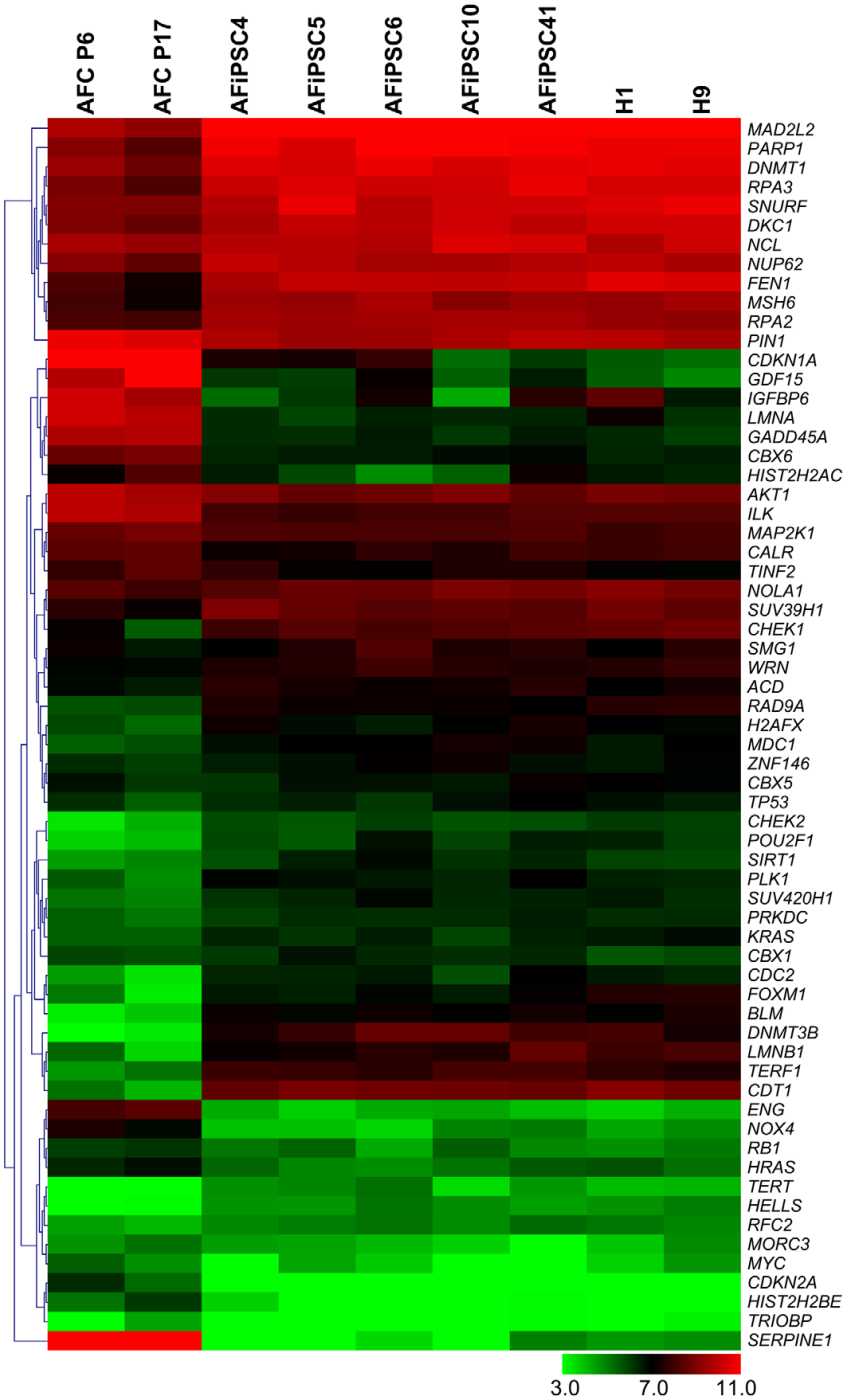
Expression of senescence-associated genes in primary AFCs and AFiPSCs. Heatmap depicting significantly differentially expressed senescence-associated genes in the union of AFiPSCs at approximately passage 20 compared to primary AFCs at passage 17 (AFC P17) (cut off: fold change ≥1.5 or ≤0.667 and FDR-adjusted P-Values for differential expression <0.05). The heatmap is colored by LOG2 average expression signals according to the color key on the bottom. Genes and samples were clustered by similar expression patterns using Eucledian distance measure.

To investigate the effect of reprogramming on bypassing senescence observed in primary AFC cultures (Figure 1A-II, -III), we analyzed the expression of senescence and telomere-associated genes in young primary AFCs (P6) and senescent AFC (P17) compared to AFiPSC lines (approximately P20). From a list of 116 senescence-associated genes (Table S4) derived from the Gene Ontology database [35], including those described by Vaziri et al. [45], we identified 64 genes as significantly differentially expressed in AFCs at passage 17 compared to the union of all AFiPSC lines (Figure 5). Of these, telomere-associated genes and genes involved in regulating the cell cycle, e.g. *MAD2L2*, *PARP1*, *RPA3*, *DKC1*, *MSH6*, *CHEK1*, *PLK1*, *POU2F1*, *CDC2*, *BLM*, *WRN*, *DNMT1*, *DNMT3B*, *LMNB1*, and *CDT1*, were down-regulated in primary AFCs compared to AFiPSCs and ESCs. In contrast, *PIN1*, *LMNA*, *GADD45A*, *CBX6*, *NOX4*, *ENG*, *HIST2H2BE*, *CDKN2A*, *CDKN1A*, *GDF15* and *SERPINE1*, among others, were up-regulated in primary AFCs compared to AFiPSCs and ESCs.

### Activation of a common ESC-like core transcriptional regulatory network in AFiPSCs and FiPSCs

**Figure 6:**
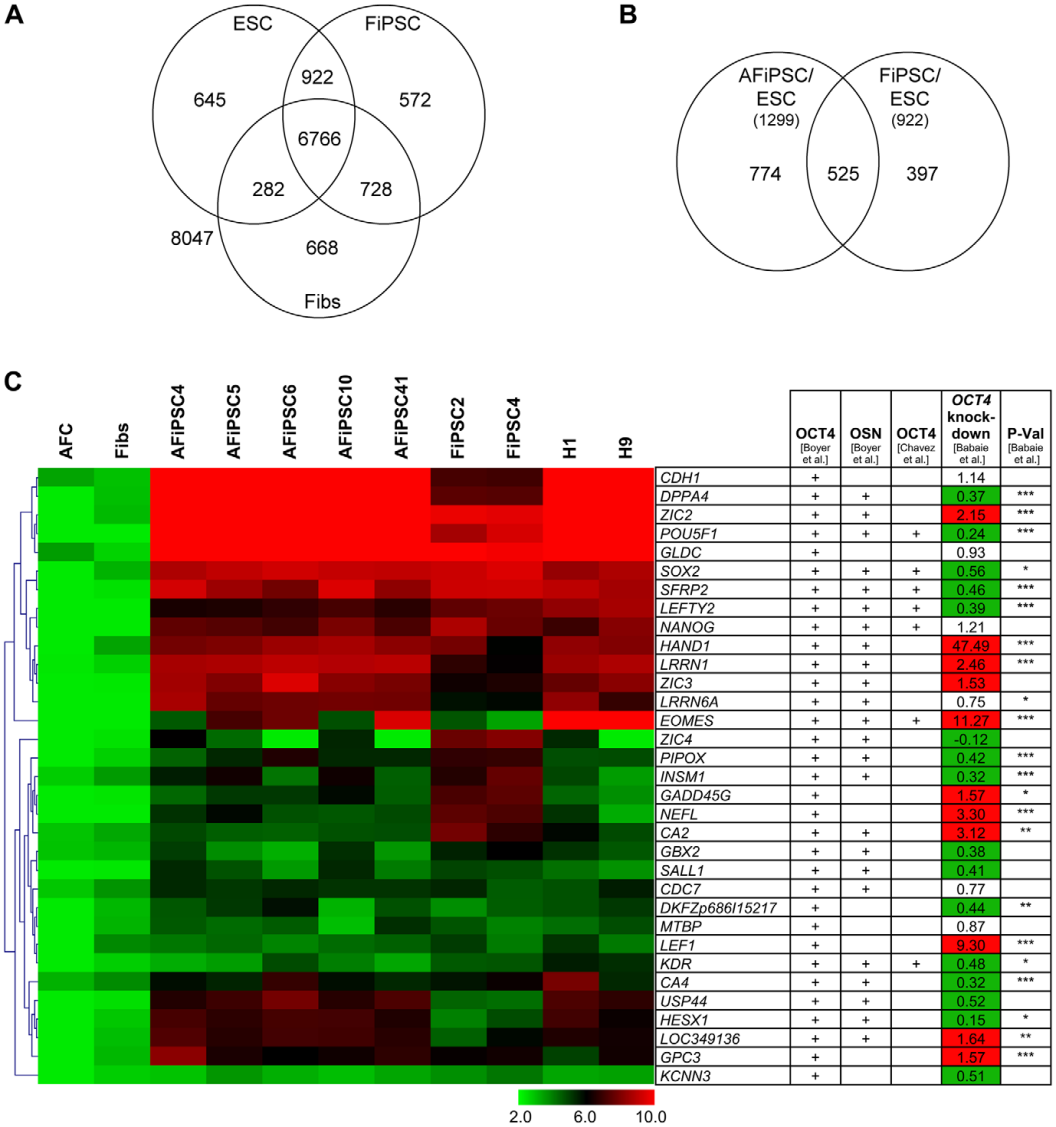
Activation of a common ESC-like core transcriptional regulatory network in AFiPSCs and FiPSCs. (A) Venn diagram analysis comparing FiPSCs, ESCs and the parental fibroblast cell line HFF1 (Fibs). (B) Direct comparison of the ESC/AFiPSC (Figure 4C) and ESC/FiPSC (Figure 6A)-derived self-renewal signature gene lists. The overlap of 525 genes expressed in all analyzed pluripotent cells (AFiPSCs, FiPSCs, ESCs) represents the core self-renewal signature. (C) Of these 525 self-renewal-associated genes, those, bound by OCT4 or simultaneously by OCT4, SOX2 and NANOG as determined by ChIP-chip analyes [46], [47], are depicted in the heatmap as LOG2 average expression signals. The heatmap is colored according to the color key on the bottom. Genes and samples were clustered by similar expression patterns using Eucledian distance measure. The table on the right identifies each gene to be bound by either OCT4 or by OCT4, SOX2 and NANOG (OSN) and shows expression changes upon siRNA-mediated *OCT4* knockdown in ESC line H1, including the differential expression P-value (P-Val) [39].

In order to narrow down the self-renewal and pluripotency signature gene list obtained by comparing global gene expression patterns of AFCs, AFiPSCs and ESCs (1299 genes, Figure 4C), we compared the same ESC samples with FiPSCs and the respective parental fibroblast line HFF1 (Fibs) (Figure 6A, the entire gene lists are presented in Table S5) [31]. Using the resulting equivalent self-renewal and pluripotency gene signature, we could detect the overlap between the two self-renewal and pluripotency gene lists derived from the separate analyses (AFiPSCs/ESCs: 1299 genes in the self-renewal/pluripotency signature, FiPSCs/ESCs: 922 genes in the self-renewal/pluripotency signature). This revealed 525 genes expressed in common in all our pluripotent cell types (AFiPSCs, FiPSCs and ESCs), highlighting their role in maintaining self-renewal and pluripotency (Figure 6B, the corresponding gene list is presented in Table S6). To gain further insight into the gene regulatory network (GRN) that induces and maintains pluripotency in AFiPSCs and FiPSCs and to define distinct functions of the 525 core self-renewal-associated genes in the undifferentiated embryonic stem cell state, we identified the overlap of these 525 genes with the list of genomic regions bound by either OCT4 alone or by OCT4, SOX2 and NANOG as identified in human ESCs by ChIP-on-chip analyses [46], [47]. This, in turn, revealed a subset of genes expressed in all of our pluripotent cell lines, which are part of an ESC-specific transcriptional regulatory network, including, for example, *POU5F1*, *SOX2*, *NANOG*, *DPPA4*, *LEFTY2* and *CDH1* (Figure 6C). To emphasize the established role of all the heatmap-listed genes in the regulation of the tightly controlled balance between the undifferentiated, self-renewing, pluripotent versus the differentiated ESC state, we combined the heatmap data in Figure 6C with gene expression data derived from siRNA-mediated *OCT4* knockdown in ESCs [39].

### The LARGE Principle of Cellular Reprogramming

**Figure 7:**
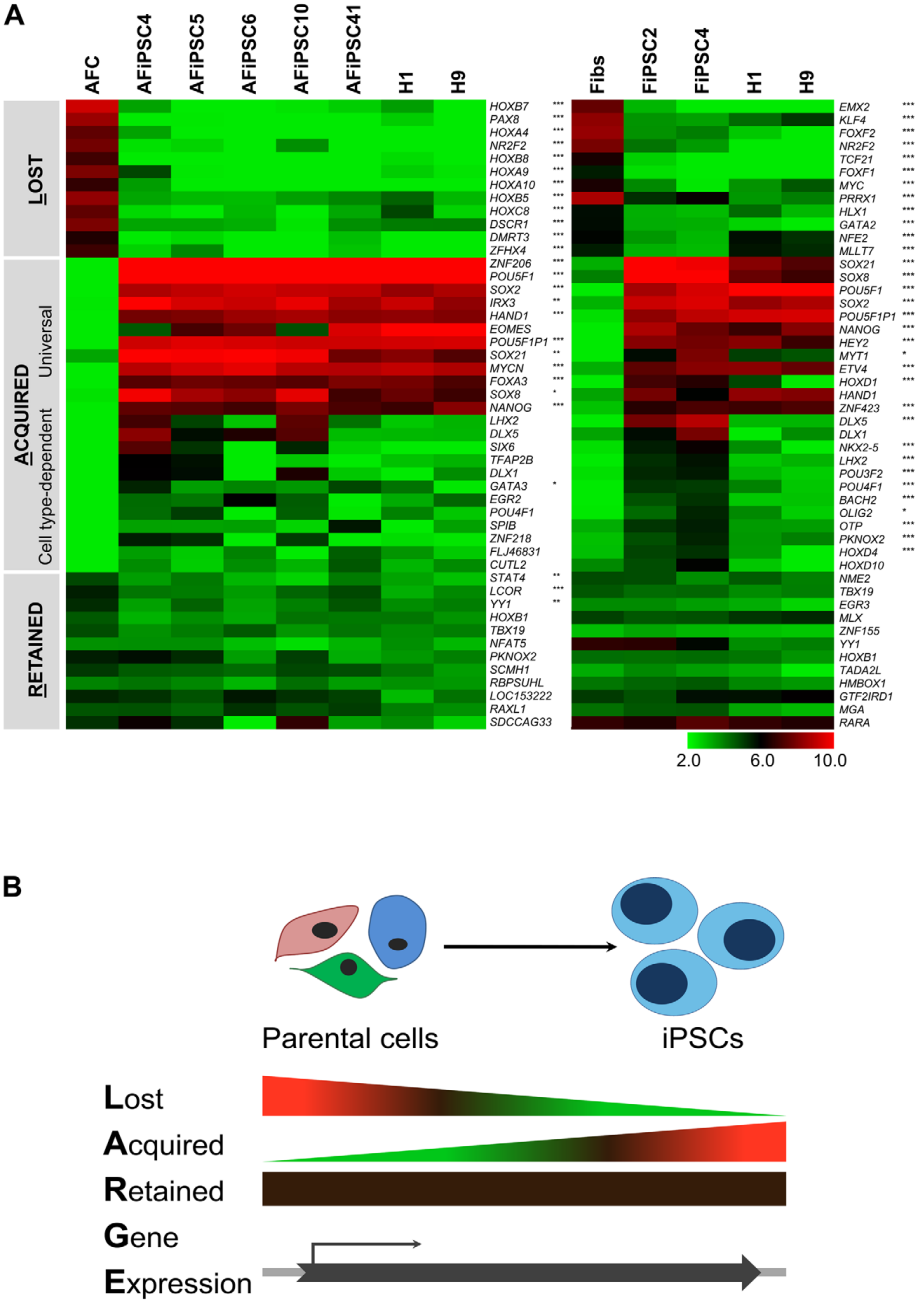
The LARGE Principle of Cellular Reprogramming. (A) To illustrate the concept of LARGE (Lost, Acquired, Retained Gene Expression), the top 12 genes with transcription factor activity and either low (LOST), high (ACQUIRED) or unchanged (RETAINED) expression values of iPSCs relative to the parental cell line were selected from the various gene expression signatures of AFCs, AFiPSCs, ESCs, Fibs and FiPSCs (Figures 4C and 6A). Significantly up-regulated or down-regulated gene expression in AFiPSCs compared to AFCs or FiPSCs compared to Fibs is indicated by asterisks (*) FDR-adjusted P-Value <0.05, **) FDR-adjusted P-Value <0.01, ***) FDR-adjusted P-Value <0.001). (B) Schematic diagram of the LARGE Principle of Cellular Reprogramming.

What can be gleaned from the global gene expression analyses presented here but also from other iPSC-based transcriptome analyses [48], [49], is that induction of pluripotency is associated with the transcriptomes of the parental cells shifting towards a distinct ESC-like state, irrespective of the cell source. More precisely, for a distinct set of genes, which are expressed in the parental cell line, expression is lost (L), whereas the expression of another group of genes is acquired (A) in the process of iPSC generation. In turn, the expression of a third set of genes, detectable in the parental cells, is retained (R) in the corresponding iPSCs. We refer to this as the LARGE (Lost, Acquired, Retained Gene Expression) Principle of Cellular Reprogramming. Also referring to other studies [48]–[50], we propose that these particular LARGE patterns are the key to understanding similarities and differences between iPSCs and ESCs and their parental cell lines on the one hand as well the heterogeneity of different iPSC types on the other. As transcription factors normally influence gene expression of several downstream targets and, thus, are likely to play a fundamental role in this concept, we used gene expression patterns of transcription factors to illustrate the LARGE concept. For this purpose, we made use of the data from the Venn diagram analyses of AFCs/AFiPSCs/ESCs and Fibs/FiPSCs/ESCs (Figures 4A and 6A, Tables S3 and S5). For each of the Lost (genes expressed in donor cells, but not in iPSCs), Acquired (genes expressed in iPSCs, but not in the donor cells) and Retained (genes expressed simultaneously in donor cells and iPSCs, excluding genes of the house keeping gene signature) Gene Expression sets, we arbitrarily picked out genes associated with the Gene Ontology term for transcription factor activity (GO0003700) [35]. Of these, the 12 transcription factors with the lowest (Lost), highest (Acquired) or least varying (Retained) expression change, when comparing AFiPSCs or FiPSCs with the corresponding parental cells, are depicted in the heatmaps in Figure 7. As a result, the group of lost transcription factor gene expressions included, for example, *HOXB7*, *HOXA9*, *HOXA10*, *PAX8*, *DSCR1*, *MYC* in AFiPSCs and *EMX2*, *FOXF2*, *FOXF1*, *MYC*, *KLF4* in FiPSCs. The acquired gene expression set can be further divided into two groups on the basis of present or absent overlaps between the two analyses for AFiPSCs and FiPSCs: those, which are universally acquired self-renewal genes present in both, AFiPSCs and FiPSCs, or, more generally, in all pluripotent iPSCs (e.g. *POU5F1*, *SOX2*, *NANOG*), and those acquired gene expressions, which are rather iPSC type-dependent (e.g. *SIX6*, *EGR2* (AFiPSCs) or *PKNOX2*, *HOXD4*, *HOXD10* (FiPSCs); *DLX5* (AFiPSCs & FiPSCs)). The retained gene expression sets included genes like *PKNOX2* (AFiPSCs); *HMBOX1*, *MGA* (FiPSCs) or *RAXL1* (AFiPSCs & FiPSCs).

## Discussion

### Ground state pluripotency of AFiPSCs

We have shown that cellular reprogramming of primary AFCs results in a fully pluripotent iPSC type, which is in line with recent publications [15], [29], [30]. We have further demonstrated that AFiPSCs are, like ESCs, at an early developmental state, in which they are not only capable of forming derivatives of the three embryonic germ layers but also of the extraembryonic trophoblast lineage. This acquisition of key ESC characteristics during cellular reprogramming should be beneficial for the application of AFiPSCs in basic and applied research, although it could be argued that the teratoma formation ability acquired by AFiPSCs hampers their use in cell replacement therapies. Yet, this is a feature of all kinds of iPSCs and ESCs, which still hold a lot of promise in this respect. Presumably, ways will be found to exploit the full differentiation potential of iPSCs while circumventing tumor formation risks, for instance, by developing accurate strategies to sort out differentiated cells of interest from potential tumorigenic stem cells.

### Cellular reprogramming bypasses senescence of bulk primary AFCs

One of the great advantages of AFiPSCs over their bulk primary counterparts for any desirable application is their acquisition of the ability to propagate indefinitely. The data presented herein suggest, that this phenotypically rejuvenated appearance of AFiPSCs is based on a gene regulatory network, which averts or at least markedly delays the onset of senescence. This is based on the fact that primary AFCs and AFiPSCs and ESCs exhibit opposing expression patterns related to a large number of senescence-associated genes. In particular, we could detect high expression levels of various cell cycle and telomere elongation-associated genes, such as *MAD2L2*, *PARP1*, *RPA3*, *DKC1*, *MSH6*, *CHEK1*, *PLK1*, *POU2F1*, *CDC2*, *LMNB1* and *CDT1*, as well as *TERT* itself in AFiPSCs in contrast to primary AFCs. The p53/p21 pathway plays a pivotal role in inducing and maintaining senescence [51]. Accordingly, mRNA levels of several p53 target genes, which are known to be up-regulated in senescent cells [52]–[54], e.g. *CDKN1A* (p21), *GDF15*, and *SERPINE1*, were strikingly elevated in primary AFCs compared to AFiPSCs and ESCs. In contrast, low level gene expression of *DNMT1* and *DNMT3B* were detected in bulk primary AFCs, whereas these genes are significantly up-regulated in AFiPSCs. Besides their function in establishing and maintaining CpG methylation patterns during embryonal development, they are also known to repress *CDKN1A* transcription in opposition to and potentially independent of p53 [54], [55]. Hence, it could be anticipated that high expression levels the *DNMTs* may repress *CDKN1A* and, thus, senescence in AFiPSCs. Taken together, there is evidence that senescence is bypassed upon the activation of a self-renewal and pluripotency program in reprogrammed AFCs, which is in line with our previous findings [31]. However, further studies are needed to assess the actual ability of AFiPSCs to restore telomere restriction fragment length to an ESC level, a subject of controversial discussion in the iPSC field [45], [56], [57].

### The LARGE Principle of Cellular Reprogramming and ESC-specific gene expression signatures

Mechanistic and functional aspects of cellular reprogramming in general, and of AFCs in particular, can be highlighted on the basis of the presented comparative transcriptome analyses of AFiPSCs, ESCs (H1, H9) and FiPSCs and the corresponding parental cell lines. For the different iPSC types we have identified genes, the expression of which are either lost (L), acquired (A) or retained (R) upon the induction of pluripotency. We refer to this as the LARGE (Lost, Acquired and Retained Gene Expression) Principle of Cellular Reprogramming. Some of these gene expression patterns, including several signature genes, are discussed.

The donor cell (AFC)-specific gene signature contains putative immune-suppressive factors, such as *CD59*, *TNFSF10*, and *NT5E* (CD73) [58]–[60], which are likely to contribute to the immune-privileged characteristics of primary AFCs [14]. Their expression is lost upon reprogramming. Whether this affects potential therapeutic applications of AFiPSCs compared to primary AFCs remains to be elucidated. Interestingly, active gene expression, which is lost upon reprogramming, could also be observed for *MYC* (AFiPSCs & FiPSCs) and *KLF4* (FiPSCs). This supports the idea that the main function of KLF4 and c-MYC in the process of reprogramming is to accelerate or enhance the efficiency by increasing a balanced cellular proliferation, while in pluripotent cells they seem to be dispensable [61]–[63].

Among the expressed genes, which are universally acquired during reprogramming processes, independent of the cell source, are key pluripotency-regulating factors, such as *POU5F1*, *SOX2* and *NANOG*. These establish a core gene regulatory network essential for maintaining self-renewal and pluripotency [46]. Yet, at the same time, expression of genes known to be involved in differentiation and development, such as *EOMES* and *HAND1*, are acquired [39], [47], [64]. These genes are direct targets of OCT4, SOX2 and NANOG [46]. They are up-regulated upon *OCT4* knockdown, and, therefore, negatively regulated by OCT4 [39]. Yet, we have observed low level expression of some developmental-related genes in all our pluripotent cell types and also in repositories of ESC and iPSC-related microarray data (e.g. http://amazonia.transcriptome.eu/temp/histo_7ebe096c97857d933b94cd30a6a120cf.png) [65]. It is conceivable, that this observation is an artefact of certain cell culture conditions or of spontaneously differentiating cells present in iPSC cultures. If, however, these genes are indeed expressed at moderately low levels in pluripotent cells, it would be worth investigating, whether this is due to distinct epigenetic marks on the promoters of these genes, similar to the concept of bivalent chromatin structures, which mark poised stem cell genes [66]. Furthermore, amongst the expressed genes, which are acquired in a cell type-dependent manner during cellular reprogramming, are those implicated in developmental processes, for example, *SIX6*, *EGR2*, *HOXD4*, *HOXD10*, *PKNOX2 and DLX5* [67]–[71]. Likewise, the list of Retained genes in both, AFiPSCs and FiPSCs, included transcription factors, such as *RAXL1*, which is also involved in the regulation of developmental processes [72]. Persistent gene expression has already been reported to contribute to differences between various iPSC types and ESCs [48] and may result in variable differentiation behaviours of iPSCs, regardless of which reprogramming technique was applied [50]. Therefore, the impact of such active, developmental genes on the ability to maintain the pluripotent state and on directed differentiation processes of different iPSC types deserves further investigation.

It is tempting to speculate, that the expression of some of the distinct signature genes are due to viral integrations into the target cell's genome. However, since these signature gene expressions were identified as a result of a group-wise analysis of several AFiPSCs/FiPSCs lines versus the parental cell lines and ESCs, they are unlikely to be attributable to clone-specific viral integrations in most cases. A probable explanation could be that these gene expression patterns are due to an incomplete erasure of epigenetic imprints in iPSCs depending on the nature of chromatin modifications of the original cell type, or in other words, a kind of cell type-specific epigenetic memory [48], [49]. However, viral integrations are probably the cause of the partially inconsistent gene expression patterns observed in the LARGE heatmap (Figure 7). In order to identify actual effects of viral integrations on the host cell genome and to avoid genomic alterations, the generation and comparative characterization of non-viral iPSCs, particularly from human AFCs, still remains.

In addition to the above-mentioned results of our LARGE analysis, we identified ESC-specific genes in the Venn diagrams, including, for example, *PRDM14*, *WNT3A* and *GSC*. *PRDM14* has been implicated in maintaining the undifferentiated ESC state [73]. In contrast, *WNT3A* and *GSC* are primitive streak/mesendoderm markers known to regulate developmental processes [43]. These genes distinguish our AFiPSCs and FiPSCs from ESCs, thus implying incomplete reprogramming and emphasizing general differences between ESCs and iPSCs despite the acquisition of the ESC phenotype in both iPSC types. Follow-up studies should be designed to identify functional consequences of this observation.

### Conclusion

Both, primary AFCs, in particular stem cell-like subpopulations of primary AFCs, as well as AFiPSCs are considered to be valuable for the application in basic and applied research. Taken together, our results propose that, for these purposes, cellular reprogramming of AFCs is beneficial as it represses senescence and leads to a phenotype very similar, though not identical, to ESCs. These findings are even more significant, considering that due to the presence of fetal stem cells within bulk primary AFCs, amniotic fluid seems to be a very suitable source of cells for the realization of non-integrating reprogramming strategies. Yet, as a main result of this study, we identified gene expression signatures and LARGE patterns for different types of iPSCs, corresponding parental cells and ESCs. Two conclusions can be drawn from this. First, this kind of comparative transcriptome analysis should be extended integrating iPSC lines derived from several distinct cell sources and generated using various reprogramming techniques, as it would aid in enhancing our meagre understanding of mechanisms underlying cellular reprogramming. Secondly, the functional relevance of such distinct expression patterns, especially of AFCs, AFiPSCs and ESCs, will have to be investigated profoundly in order to estimate limitations and to exploit the full potential associated with putative future utilization of amniotic fluid-derived cells.

## Supporting Information

Table S1 List of primers used for qRT-PCR and DNA fingerprinting analyses.
(0.03 MB XLS)

Table S2 List of antibodies used for immunofluorescence stainings.
(0.02 MB XLS)

Table S3 Complete gene lists reported in the ESC/AFiPSC/AFC-Venn diagram (Figure 4C). Spreadsheet 1 a–c: Self-renewal signature, 1299 genes, (a) gene list (GL), (b) DAVID functional annotation chart (DFACha), (c) DAVID functional annotation clustering (DFAClu). Spreadsheet 2 a–c: ESC-specific genes, 257 genes, (a) GL, (b) DFACha, (c) DFAClu. Spreadsheet 3 a–c: AFiPSC-specific genes, 555 genes, (a) GL, (b) DFACha, (c) DFAClu. Spreadsheet 4 a–b: Housekeeping genes, 6934 genes, (a) GL, (b) DFACha. Spreadsheet 5 a–c: Overlap ESCs and AFCs, 125 genes, (a) GL, (b) DFACha, (c) DFAClu. Spreadsheet 6 a–c: Donor cell memory, 350 genes, (a) GL, (b) DFACha, (c) DFAClu. Spreadsheet 7 a–c: Donor cell-specific genes, 665 genes, (a) GL, (b) DFACha, (c) DFAClu.
(5.87 MB XLS)

Table S4 Senescence-associated genes. List of 116 senescence-associated genes derived from the Gene Ontology database [35], including those described by Vaziri et al. [45]. These genes served as input for the differential gene expression analysis between AFCs (P17) and the group of all AFiPSC lines.
(0.04 MB XLS)

Table S5 Complete gene lists reported in the ESC/FiPSC/Fibs-Venn diagram (Figure 6A). Spreadsheet 1 a–c: Self-renewal signature, 922 genes, (a) GL, (b) DFACha, (c) DFAClu. Spreadsheet 2 a–c: ESC-specific genes, 645 genes, (a) GL, (b) DFACha, (c) DFAClu. Spreadsheet 3 a–c: FiPSC-specific genes, 572 genes, (a) GL, (b) DFACha, (c) DFAClu. Spreadsheet 4 a–b: Housekeeping genes, 6766 genes, (a) GL, (b) DFACha. Spreadsheet 5 a–c: Overlap ESCs and Fibs, 282 genes, (a) GL, (b) DFACha, (c) DFAClu. Spreadsheet 6 a–c: Donor cell memory, 728 genes, (a) GL, (b) DFACha, (c) DFAClu. Spreadsheet 7 a–c: Donor cell-specific genes, 668 genes, (a) GL, (b) DFACha, (c) DFAClu.
(6.57 MB XLS)

Table S6 Complete list of 525 core self-renewal genes as reported in Figure 6B. Spreadsheet a–c: Core self-renewal signature, 525 genes, (a) GL, (b) DFACha, (c) DFAClu.
(0.67 MB XLS)
